# Notes of the Week

**Published:** 1921-06-25

**Authors:** 


### NOTES OF THE WEEK.

### IRELAND AND HER HOSPITALS.

All eyes are turned to Ireland where, as we go to press, the King is performing the historic ceremony of opening the first Ulster Parliament in Belfast. Hospital activities in Belfast are not the least important of those that have helped to build up the reputation of this famous seaport, though the number of beds per thousand of the population has not been equal to that of Dublin. The latest figures, however, show that the number of hospital beds per thousand of the population for the whole of Ireland is rather more than the average for England and Wales, excluding London, though it is less than the average for Scotland. Ireland distinguishes herself in her adoption and development of the system of workpeople's contributions; no less than 23.6 per cent. of the joint total ordinary income in 1919 of some half-dozen Irish hospitals was derived from this source. To Belfast too belongs the distinction of possessing a hospital, the Royal Victoria, whose new buildings, opened by King Edward and Queen Alexandra in 1903, were held at the time to embody a revolution in hospital design. They included the Plenum system of ventilation, then new to that country, though it had been employed by the same architect, Mr. William Henman, in the Birmingham General Hospital, and was in considerable use in hospitals in the United States.

### MR. WALTER ALVEY'S SUCCESSOR.

As we appear a meeting of the Charing Cross Hospital authorities is being held at which the confirmation of the appointment of a successor to the late Mr. Walter Alvey will be made. We learn that Mr. Philip Inman has been selected out of 512 candidates, and that he has sent in his resignation to the Chairman of the Hospital Saturday Fund. All who have followed Mr. Inman's work for this Fund and for the better organisation of the support of the workpeople and their employers in the interests of hospitals will regret the loss which his transference from the Saturday Fund to work on behalf of one individual hospital must mean to Metropolitan hospitals as a whole. There is no doubt, however, that Charing Cross Hospital is to be congratulated. Mr. Inman, who is a Yorkshireman, and by training a chemist, has ideas, energy and organising power. This is, we think, in London at least, the first time that a secretary of a central fund has become the chief lay officer of one of the hospitals, although we remember that Mr. Owthwaite, when secretary of the City of London Lying-In Hospital, did important work for the Hospital Sunday Fund.

### A JUBILEE AT 800 YEARS.

On June 21, 1871, Queen Victoria, with whom were the then Prince and Princess of Wales, opened the new St. Thomas's Hospital buildings on its present site. On Tuesday last this great hospital, which has been going on for about 800 years, celebrated this jubilee by the presence of its President, H.R.H. the Duke of Connaught, who distributed the prizes to the students of the medical school, opened the new Sherrington School of Physiology, and afterwards was present at a garden-party in the grounds, when the nursing staff presented him with a cheque for £2,120 which they had collected to help their Alma Mater in her present serious financial difficulties. The speeches of Sir Arthur Stanley and Sir Cuthbert Wallace were of particular interest, reviewing as they did the proceedings fifty years ago, the development of the hospital to meet the position after the war, and the growth of the medical school. Sir Arthur laid stress on the splendid work accomplished by the late treasurer, Sir James Wainwright, to whose foresight the sound pre-war financial position was due. Present conditions have changed this; the expenditure has risen from £76,000 to £172,000, the hospital has a loan of £83,000 and a bank overdraft of £20,000. Ninety beds are closed, and the appeal for £100,000 has up to date realised only £13,500. No wonder that the hospital's position is considered serious. We are glad to note that efforts are being made to organise collections among firms; it is in this direction and by patients' payments that the financial salvation of our great hospitals is held to lie.

### HOSPITAL MANAGERS IN CONFERENCE.

The annual meeting of the British Hospitals Association will take place at the Manchester Royal Infirmary on Friday, July 1, at 3 p.m. The meeting will be one of special interest, as besides the usual formal business a discussion will take place on the recommendations of Lord Cave's Committee. In particular, we understand, will the question of co-ordinated appeals and the position of the approved societies be considered. It behoves all who have the interests of their institutions at heart to make a point of attending this meeting. The last occasion on which the British Hospitals Association met in Manchester was in September 1911.

### THE RECEPTION OF THE CAVE REPORT.

News is now available concerning the reception of the Cave Commission's Report on the position of the voluntary hospitals. In the *Yorkshire Post* certain opinions have been published by those qualified to speak. The chief doubt appears to be whether the sum of £250,000 for possible extensions is additional to, or part of, the million named in the report. It is felt that it should be an addition if the relief recommended is to be adequate. Mr. Charles Lupton, who lately retired from the Chairmanship of the General Infirmary, Leeds, seems to think that London and the South of England have not shouldered the burden as well as the people of Leeds are trying to do. Mr. A. J. Atkinson, Chairman of the Committee of Hull Royal Infirmary, thinks that the grant will need to be continued for three years at least. Mr. Fred Knowles, Secretary of the Manchester Saturday Fund, thinks the proposed grant of £250,000 somewhat small. Mr. Arthur Wilkinson, Secretary of the York Workpeople's Hospital Fund, welcomed many of the recommendations, especially that of a committee to consider local hospital needs. Mr. W. Weir, President of the Northumberland Miners' Association and a Governor of Newcastle Royal Infirmary, favoured the report because he was a believer in State aid without State administration. Such aid, he thought, would encourage and not diminish working-class support of hospitals. The Scotch papers are gratified at the relatively favourable position of the Scotch hospitals shown in the report. If the Glasgow and Edinburgh hospitals are in difficulties, the Glasgow Royal Infirmary succeeded last year in raising the annual contributions from public works from £16,600 to almost £31,000.

### A HOSPITAL PIONEER.

An interesting phase in hospital history was commemorated when the portrait of Sir W. J. Smyly was lately presented to the Rotunda Hospital, Dublin, of which Sir William was Master from 1889 to 1896. Dr. E. Hastings Tweedy, Past Master, made the presentation, and said that the theatre built on Sir William's plans was the first of the modern type now universally adopted. It was he who had adopted the early methods of modern surgery; to him the hospital owed its modern gynæcological wing, given by the late Thomas Plunket Cairnes. Again, through Sir William the first fully-trained nurse, Miss Sarah Hampson, was appointed Lady Superintendent, and the modern standard of training was introduced. The portrait is to be hung in the board-room in recognition of the work of a Master who was also a pioneer in his day.

### A RECORD GIFT AND A NEW POLICY.

The Committee of the Bristol Royal Infirmary has decided to place in the entrance-hall a tablet recording the "largest gift" ever received by the hospital. The inscription reads: "This is to record that in 1920 the President, Henry Herbert Wills, J.P., generously presented securities to the nominal value of £105,070 to the Bristol Royal Infirmary. This is the largest gift the Bristol Royal Infirmary has ever received, and will produce £5,000 per annum." The institution has received this sum absolutely, because the proposed amalgamation scheme fell through. Other interesting, though necessarily smaller, gifts have been the gift of the cost of the annual report, and, through the influence of Mr. Ellis C. Smith (Mr. W. E. Budgett's successor in the secretaryship), of all the stout and beer required by the hospital; this latter gift has been made by Messrs. Davenports, Ltd., of London. An appeal is being issued to pay off the remainder of the overdraft. Subscribers' notes, we are glad to see, are to be abolished at the end of the year. A weekly charge upon patients of one guinea toward the cost of maintenance is to be established and an almoner to be appointed. The medical staff offers to treat free all patients found by the almoner to be able to pay no more than the cost of their maintenance, but patients paid for by public authorities or approved societies will be treated by the staff in return for an agreed percentage of the gross sum received by the Royal Infirmary. This percentage is to be paid into a fund to be administered by the honorary medical staff.

### A HOSPITAL CHAIRMAN'S BEQUESTS.

The late Mr. T. Fielding Johnson, J.P., senior vice-president and ex-chairman of the Leicester Royal Infirmary, bequeathed generous legacies to Leicestershire institutions. The Leicester Royal Infirmary receives £1,000, and the Children's Hospital £500, the Leicester Maternity Hospital £1,000, the Faire Hospital, Leicester, £500, the Leicester Institution for the Blind £200, the Wycliffe Society for Helping the Blind £100, the Leicester District Nursing Association £200, the Leicestershire County Nursing Association £200, and the Charnwood Forest Convalescent Home £250. Other bequests include £250 to the Leamington Home for Incurables, £500 to the Leicester Great Meeting, £200 to the Free Christian Church, Narborough Road, £200 to the Domestic Mission in connection with the Great meeting, £150 to the Dr. Barnardo's Homes, and £100 to the Trustees of the Wyggeston Grammar School Foundation. The total bequests to institutions thus amounts to £5,350. In addition to the above bequests, his generosity to the Leicester Royal Infirmary and Children's Hospital has often been mentioned in The Hospital, and his munificent gift of the site for the University College and Wyggeston Schools was recently referred to in these columns.

### CO-ORDINATION IN GLOUCESTER.

Our readers will recall the account given in The Hospital of the scheme for co-ordinating hospitals and medical treatment in Gloucestershire, which Dr. Middleton Martin, the medical officer of health, largely planned, and which received the blessing of the different medical and institutional bodies in the area. The approval of the Ministry of Health was eventually gained, and a beginning has now been made with the new centres which, when established, will be accessible in every four-mile radius through the county. The Joint War Committee of the Red Cross and the Order of St. John made at the start a grant of £7,000 towards the scheme, which is controlled by a committee representing all the bodies concerned, under the chairmanship of Mr. W. F. Hicks-Beach. The eleven cottage hospitals are to have out-patient departments attached or developed; forty-one out-centres are to be established, staffed by local practitioners, with periodic visits from consultants from the general hospitals. The various clinics for consumptives, school children, and cases of venereal disease are included in the scheme. In the main it co-ordinates existing agencies, for the new centres will be like local surgeries or dispensaries, staffed by local men. The most remarkable fact is that the County Council, the Government departments, the medical profession, and the hospitals have all been brought into accord.

